# Supplementary material for: Causal Relationship between Aging and Anorexia Nervosa: A White-Matter-Microstructure-Mediated Mendelian Randomization Analysis
Source: Biomedicines. 2024 Aug 16;12(8):1874. doi: 10.3390/biomedicines12081874 (PMC11351342; doi:10.3390/biomedicines12081874)
Supplement: Supplementary file 1 [file biomedicines-12-01874-s001.zip › Supplementary Figure/Figure S9/Leaveoneout plot of MR analysis for ubm-b-1676 to AN.pdf]

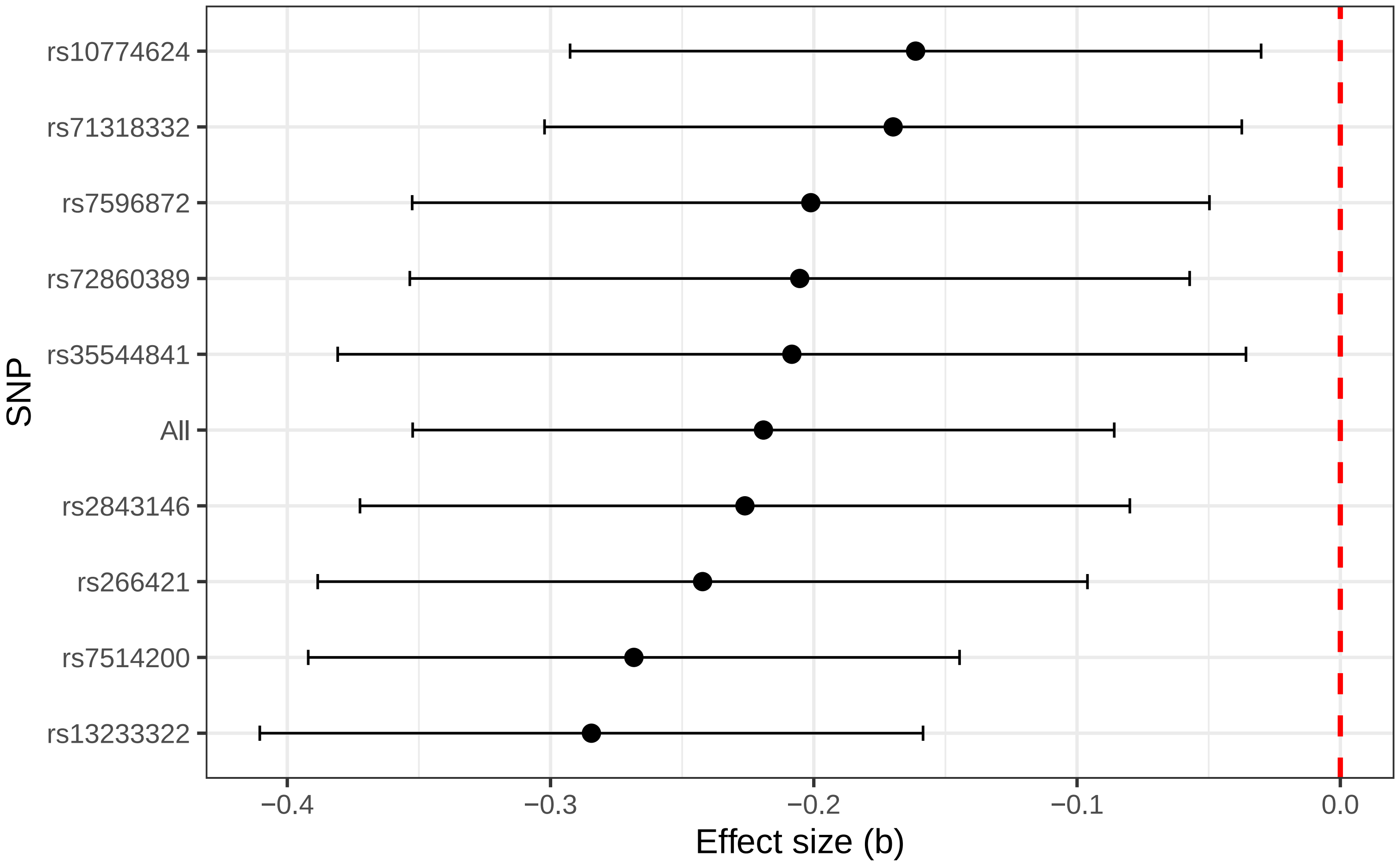
SNP
rs10774624
rs71318332
rs7596872
rs72860389
rs35544841
All
rs2843146
rs266421
rs7514200
rs13233322
−0.4
−0.3
−0.2
−0.1
0.0
Effect size (b)
